# ResTransUNet: a dual-encoder hybrid network for automated liver segmentation in CT scans

**DOI:** 10.1038/s41598-026-46342-y

**Published:** 2026-04-01

**Authors:** Yuan Wang

**Affiliations:** The School of Medicine, Medical Sciences and Nutrition, University of Aberdeen, Scotland, AB24 3FX UK

**Keywords:** Liver segmentation, Deep learning, Transformer, Convolutional neural networks, Medical image analysis, CT scans, Hybrid network, ResTransUNet, Attention mechanism, U-Net, Computational biology and bioinformatics, Engineering, Mathematics and computing

## Abstract

Precise delineation of the liver is essential for effective diagnosis and treatment planning in hepatocellular carcinoma, yet current clinical practices rely heavily on manual annotation, which is both inefficient and susceptible to human error. In this work, we propose a fully automated segmentation framework, termed ResTransUNet, that leverages the complementary strengths of convolutional operations and self-attention mechanisms. The architecture introduces a bifurcated encoder design wherein spatially local features are captured through convolutional layers, while long-range dependencies are simultaneously modeled using a Transformer-based path. To bridge these parallel streams, we incorporate a feature refinement module that infuses the globally aware Transformer representations into the CNN-derived features, thereby enhancing contextual comprehension without incurring excessive computational cost. This hybrid strategy addresses the limitations commonly observed in conventional U-Net structures–particularly the inability to capture global semantics–and avoids the high complexity often associated with pure Transformer models. Extensive experimentation on the LiTS2017 benchmark reveals that the proposed model achieves superior segmentation accuracy, reporting a Dice score of 0.9535, a volumetric overlap error of 0.0804, and a relative volume difference of −0.0007. Additional validation across diverse public datasets, including 3Dircadb, CHAOS, and Sliver07, demonstrates the model’s consistent generalization and robustness, particularly in challenging scenarios involving small, fragmented, or poorly contrasted liver regions.

## Introduction

The liver, being the largest internal organ in the body, is centrally involved in metabolic control, detoxification, and other homeostatic functions^1^. Because of its intricate structure and participation in a very wide range of physiological processes, it is highly vulnerable to an exceedingly large number of pathologic conditions, including hepatocellular carcinoma, which continues to be one of the most common and fatal types of malignancies worldwide. Precise and automated segmentation of the liver from abdominal imaging modalities, viz., computed tomography (CT), is thus crucial for accurate diagnosis, volumetry, treatment planning, and surgical navigation. Conventional liver segmentation procedures, nevertheless, depend mostly on manual tracing of liver boundaries on consecutive image slices, which is time consuming in nature and prone to high inter-observer variability. The reliance on skilled radiologists for this task renders the process time-consuming and inconsistent, thereby hindering the widespread use of streamlined and reproducible diagnostic protocols. In clinical settings where the fast and accurate interpretation of imaging information is a priority, the demand for strong, automated segmentation solutions has become increasingly evident. Computer-assisted approaches have thus been explored as promising alternatives, providing scalable solutions that minimize the effort of manual intervention without compromising on clinical significance and anatomical accuracy.

In the past decades, researchers have proposed a large number of automatic liver segmentation techniques^2^. The existing techniques can be broadly divided into three main categories: classical image processing techniques^3–6^, machine learning-based frameworks^7–10^, and deep learning-based approaches^11–18^. Conventional image processing techniques–like thresholding, region growing, and level-set methods–are computationally efficient and fast^3–6^. However, they fail for difficult cases because of their low tolerance to noise, liver morphological variation, and blurry organ borders, requiring them to be only semi-automatic and with high dependence on manual parameter initialization. Machine learning-based methods are an improvement by introducing data-driven models, which employ handcrafted features to restrict segmentation processes. These approaches have been successful but constrained in their improvement in accuracy by the requirement of feature engineering and limited capacity to model high-level semantic information in generalizing to shifting data distributions^7–10^.

Deep learning, in the form of convolutional neural networks (CNNs), has revolutionized medical image analysis. Deep learning approaches eliminate the necessity for hand-designed feature extraction and have been successfully used for tasks ranging from classification and object detection to semantic segmentation. Among them, Long et al.’s^11^ Fully Convolutional Network was a significant milestone in removing fully connected layers and using convolutional operations instead to facilitate dense pixel-wise prediction. This early work opened the door for further innovation, the most prominent of which is the U-Net architecture by Ronneberger et al.^13^ that was specifically designed for biomedical image segmentation. The U-Net model, with its encoder-decoder framework and skip connections, has seen extensive use as it effectively localizes fine anatomical details without losing contextual depth. Subsequent enhancements, such as the V-Net of Milletari et al.^16^, incorporated residual learning to further improve volumetric segmentation performance, particularly in three-dimensional datasets^19^. These efforts cumulatively paved the way for modern medical image segmentation pipelines.

Despite the progress, traditional CNN-based architectures still have inherent limitations when applied to liver segmentation. Various enhancements have been proposed to mitigate these limitations. Christ et al.^20^, for example, presented a cascaded FCN architecture supported by 3D conditional random fields to improve boundary segmentation in liver and tumor areas. Kaluva et al.^21^ developed dense FCN models that improved spatial detail preservation through architectural innovations. The H-DenseUNet model introduced by Li et al.^22^ fused 2D and 3D U-Net architectures to achieve a compromise between slice-wise and volumetric features to improve tumor segmentation. Han et al.^23^ also developed a deep FCN model with 24 layers and added skip connections to fuse low- and high-level semantics effectively. Although these efforts are valuable enhancements, limitations like gradient vanishing, resolution loss due to downsampling, and category imbalance are still common issues with FCN and U-Net-based architectures. These drawbacks are particularly severe when segmentation of small or disconnected liver areas is attempted, and when images with low contrast or vague anatomical borders are processed.

In addition, the limitations of handcrafted feature dependence and weak contextual awareness motivated researchers to investigate architectures capable of modeling spatial dependencies at long ranges. The introduction of transformers to the computer vision field^24^ represented a paradigm shift in that it allowed models to represent global relationships among features. Initially conceived for sequence modeling tasks in natural language processing, transformer-based models have been effectively translated to visual domains with the benefits of improved generalization and spatial awareness. Initial uses of transformers in medical imaging mainly leveraged global self-attention, which, although effective, came at high computational costs due to quadratic complexity relative to feature map sizes. To remedy this, recent efforts have introduced local attention mechanisms with fixed computation windows, thus maintaining contextual sensitivity while improving scalability. These advancements created new opportunities for combining transformers with convolutional operations to leverage their respective strengths in local pattern recognition and global information modeling.

However, in spite of the potential shown by CNNs and transformer models separately, there is much scope for improvement in the realm of liver segmentation. Liver images in real-world clinical data tend to have high complexity in terms of unclear boundaries, irregular shapes, and fragmented areas. Such scenarios reveal the weakness of current architectures, which can fail to ensure accuracy and consistency for varied cases. As such, there is an urgent need to come up with hybrid models that are able to take advantage of the localized feature extraction of CNNs while enjoying the long-range dependency modeling of transformers. This research seeks to overcome these drawbacks by proposing a new automatic segmentation framework, ResTransUNet, which features a dual-path encoder framework assisted by a feature enhancement unit. Unlike TransUNet which applies transformers only at the bottleneck, and SwinUNet which relies purely on transformers, ResTransUNet introduces a dedicated Feature Enhancement Unit (FEU) that creates bidirectional communication between CNN and transformer pathways at every encoder stage, enabling simultaneous local-global feature refinement throughout the entire encoding process. The model aims to combine the rapid convergence and spatial accuracy of U-Net with the representational depth of transformer attention mechanisms to facilitate high segmentation performance on both common and challenging liver images.

## Related work

###  Convolutional neural network-based segmentation approaches

Early progress in medical image segmentation was largely led by classical methods such as contour-based approaches and traditional machine learning algorithms, more so for organ boundary detection and delineation applications^21,23^. Though these methods laid the foundation for computational segmentation, the advent of deep learning and specifically the invention of Convolutional Neural Networks (CNNs) represented a major shift away from rule-based and hand-engineered approaches. A seminal breakthrough was achieved with the advent of U-Net by Ronneberger et al.^13^, which introduced a U-shaped encoder-decoder framework that has become a backbone architecture for biomedical segmentation applications. Its ease of implementation, coupled with the seamless integration of skip connections to combine low-level and high-level semantic features, was largely responsible for its use and popularity in clinical image processing workflows. The impact of U-Net has spawned a profusion of derivative architectures that build upon and refine its seminal structure. Representative examples include Res-UNet^28^, HDense-UNet^22^, U-Net++^14^, and UNet3+^29^, each incorporating innovations like dense connections, residual learning, and hierarchical aggregation to enhance feature reuse, gradient propagation, and contextual encoding. These variants have shown improved performance in a wide range of medical image segmentation tasks. In the particular application of liver segmentation, specialized architectures have been designed to overcome domain-specific challenges like low contrast, irregular organ shapes, and partially obscured boundaries. SAR-UNet^30^ is a case in point by combining the Squeeze-and-Excitation (SE) block with Atrous Spatial Pyramid Pooling (ASPP) to dynamically recalibrate feature maps and capture scale-invariant representations. By using residual connections, this model also alleviates vanishing gradient issues, thereby facilitating deeper network training without compromising convergence stability or segmentation accuracy.

A number of the newer models have focused on inserting classic convolutional architectures with attention blocks in order to enhance the localization of anatomically significant areas. For instance, GCHA-Net^17^ uses a global attention block (GAM) and local attention mechanism (LAM) within a composite feature aggregation architecture in order to solve problems related to blurry liver boundaries and tumor heterogeneity. This kind of setup encourages global semantic perception as well as accurate spatial perception. Simultaneously, Eres-UNet++^31^ adds channel-wise attention and residual blocks to the baseline U-Net++ backbone that together enable the network to adaptively weight important features and mitigate the effect of class imbalance in distributions. These additions together improve segmentation results stability across anatomical cases. The application of the U-Net idea to volumetric data has collectively propelled advances in three-dimensional medical image segmentation. Architectures like 3D-UNet^15^ and V-Net^16^ are typical of this shift, employing three-dimensional convolutional kernels to leverage spatial relationships across every anatomical plane. These networks have proved quite competent in the contextual information capture across volumetric scans, thereby enhancing segmentation accuracy in datasets that need inter-slice coherence. The capacity of CNN-based models to learn hierarchical structured and meaningful representations has been the signature of their success in practice, particularly when used in clinical applications where anatomical correctness is paramount. Consequently, CNN-based architectures continue to be the leading backbone of modern segmentation pipelines owing to their computational scalability and complexity and their generalizability across imaging modalities.

### Transformer-based vision models for medical segmentation

Transformers were initially associated with the rise of machine translation tasks, whereby they were first proposed as components of the sequence-to-sequence modeling paradigm^24^. Their application in Natural Language Processing (NLP) soon spread and achieved state-of-the-art performance on a large number of language modeling tasks^32^. Inspired by this success, the research community adapted transformer architectures to visual applications, and the Visual Transformer (ViT) was proposed first by Dosovitskiy et al.^33^. It was well-balanced between classification performance and computational efficiency. Reliance on large pre-training corpora, however, was a drawback that posed a challenge in applying the model in medical imaging applications where generating labeled data is generally challenging and expensive to procure. Through the solution of the challenge in training ViT with limited data, Deit^34^ proposed a sequence of architecture and regularization techniques that facilitated successful transformer training on general-scale datasets like ImageNet. This was subsequently refined with significant advancements in the adaptation of transformer models to dense prediction tasks. In particular, the Swin Transformer^37^ proposed a hierarchical framework based on shifted window operations that maintained locality while solving the quadratic complexity problem of global self-attention. This architecture presented an interesting solution to medical image segmentation by enabling multiscale context integration and smaller computational complexity. Swin Transformer has been shown to achieve high performance on tasks such as image classification, object detection, and semantic segmentation and is therefore a desirable choice for hybrid network architecture.

Some hybrid architectures have been designed where the strengths of transformers and CNNs are combined to overcome their respective weaknesses. TransUNet^25^ is one such example which incorporates transformer-based attention modules into a U-Net-like architecture. By integrating the strengths of long-range dependency modeling in transformers with local feature encoding capabilities of convolutional layers, TransUNet enhances contextual understanding in heavy medical images. Another effective design, Swin-UNet^26^, incorporates a hierarchical vision transformer in an encoder-decoder framework. It utilizes shifted windows for efficient context aggregation and a symmetric decoder with patch expansion mechanisms to recover spatial resolution. This model shows the power of pure transformer-based models to overcome CNN locality constraints, especially in modeling far-distance spatial relations that are essential for global organ delineation. Based on this increasing collection of works, the current work adopts the Swin Transformer as a core component in a U-shaped encoder-decoder segmentation model. Several recent works have further advanced the field of hybrid medical image segmentation. Sun et al. proposed DA-TransUNet^32^, which integrates spatial and channel dual attention blocks into the TransUNet framework, enabling more precise extraction of position-specific and channel-specific features through dual attention mechanisms applied at both the encoder and skip connection levels. Pan et al. introduced MIPC-Net^33^, a boundary-focused segmentation architecture that employs a mutual inclusion mechanism between position and channel attention to enhance precise boundary delineation in medical images. Sun et al. also proposed FKD-Med^34^, a federated learning framework for privacy-aware medical image segmentation that integrates knowledge distillation to reduce communication overhead while maintaining competitive segmentation performance. Additionally, Sun et al.^35^ combines multi-scale context aggregation with selective long-range boundary refinement for challenging lesion segmentation scenarios. The addition here is with skip connections between encoder and decoder layers to preserve spatial consistency and promote feature reuse upon decoding. Through the integration of transformer-based modules into a segmentation network for volumetric medical imaging, our method aims to illustrate how contextual awareness and spatial accuracy may be enhanced together. This method aims to expand the application scope of transformer networks for medical image analysis and provide a basis for future work in transformer-convolution hybrid structures for automatic liver segmentation.

## Methodology

**Fig. 1:**
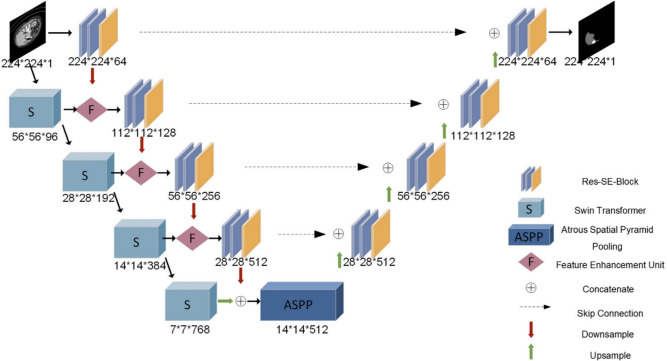
Overview of the ResTransUNet architecture. The model integrates a U-Net encoder-decoder backbone with a parallel transformer path, multiple residual and Squeeze-and-Excitation (SE) blocks, and an ASPP module.

This section delineates the proposed ResTransUNet framework’s technical design and constituents. The network follows a convolutional encoder-decoder backbone inspired by the U-Net architecture with an additional parallel stream of transformers for context enrichment. The system combines a Swin Transformer module, a specialized feature enhancement unit (FEU), and a composite loss function to achieve high-accuracy liver segmentation. All components of the model cooperate to enable the representation of local details and global dependencies, resolving common drawbacks in traditional segmentation workflows. The overall system is schematically illustrated in Fig. 1.

### Architecture

The fundamental architecture of ResTransUNet is built by combining a U-Net encoder-decoder and a transformer-based contextual encoder. While the U-Net encoder is tasked with hierarchical feature extraction, the decoder recovers spatially coherent predictions using upsampling and fusion operations. Concurrently, the transformer branch aggregates wider spatial relationships and long-range interactions and consequently enriches the encoder features with global context. The complete configuration consists of 8 residual units, 4 Swin Transformer blocks, 4 Squeeze-and-Excitation (SE) modules, 4 downsampling layers, 4 upsampling layers, and 1 Atrous Spatial Pyramid Pooling (ASPP) module. All convolution operations utilize a kernel size of $3 \times 3$, and downsampling is performed using $2 \times 2$ max pooling. The input resolution is set to $224 \times 224 \times 1$, and all operations maintain input-output spatial coherence through judicious padding and stride control. This serial combination of convolution, pooling, and nonlinear activation ultimately produces a binary segmentation mask of equal dimensions.

**Fig. 2:**
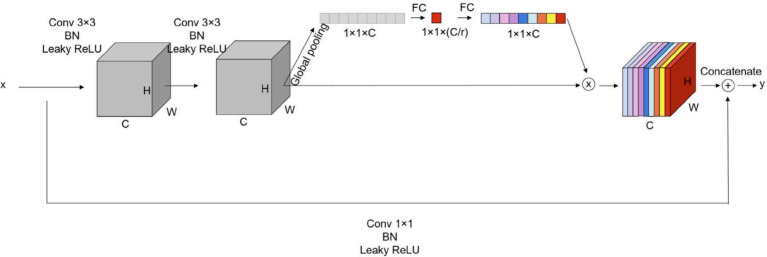
Schematic of the Squeeze-and-Excitation (SE) mechanism used after each residual block. The block performs channel-wise recalibration using global average pooling and learned gating to emphasize salient features.

Residual connections, motivated by the ideas of deep residual learning^19^, are used in all U-Net convolutional blocks to ensure effective gradient flow. The skip connections alleviate the degradation issue in deep networks and allow feature reuse across layers. Batch normalization and Leaky ReLU activations are used after convolution to stabilize learning and avoid overfitting. The Leaky ReLU is especially helpful for keeping non-zero gradients for negative activation regimes, avoiding vanishing gradient issues in deeper networks. Every residual unit (minus the first one) is supplemented with an SE block^38^ for channel-wise recalibration of feature responses. The SE module adaptively highlights informative channels in three consecutive steps: first, a global average pooling layer condenses spatial information into a channel descriptor; second, a fully connected bottleneck introduces a nonlinear transformation to produce channel-wise weights; and third, the weights are scaled and applied to the original feature maps to reinforce discriminative channels. This operation is visualized in Fig. 2, which shows the SE attention mechanism working after every residual block to distill semantic representations.

**Fig. 3:**
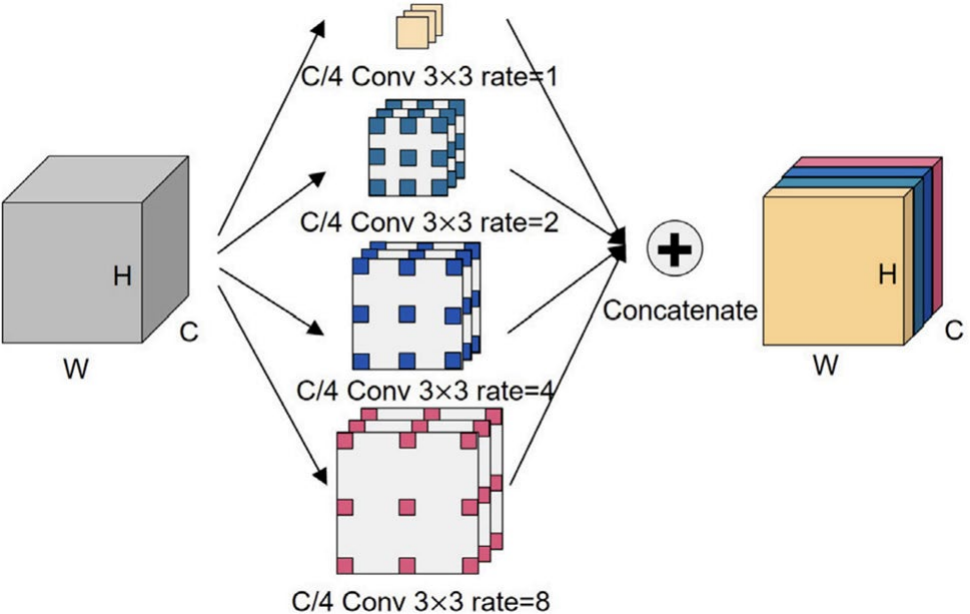
Illustration of the Atrous Spatial Pyramid Pooling (ASPP) module. It captures multi-scale context through parallel dilated convolutions with varying rates, improving segmentation of spatially diverse liver regions.

To alleviate the spatial resolution loss caused by consecutive downsampling, the architecture introduces an ASPP module as a bridge layer, illustrated in Fig. 3. ASPP extracts multi-scale context through parallel dilated convolutions with different receptive fields, allowing for the modeling of spatially varying features in medical images.

### Swin transformer block

**Fig. 4:**
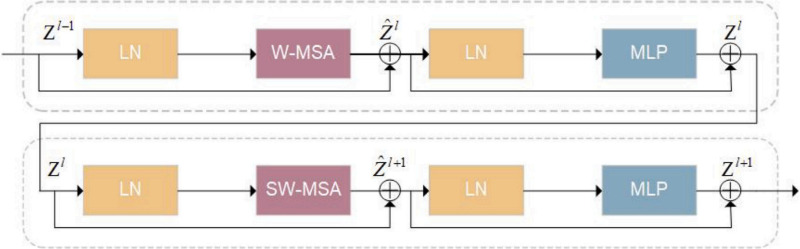
Architecture of two consecutive Swin Transformer blocks. Each block employs Layer Normalization (LN), window-based or shifted-window-based self-attention (W-MSA/SW-MSA), and a multi-layer perceptron with residual connections.

The Swin Transformer blocks incorporated into the encoder are geared to capture non-local information via hierarchical self-attention. Four of these blocks are included in the transformer pathway of ResTransUNet. In contrast to conventional multi-head self-attention (MHSA) mechanisms, which depend on full spatial resolution and global windowing, Swin Transformers are based on a window-based method with local partitioning. Every Swin block consists of Layer Normalization (LN), window-based multi-head self-attention (W-MSA), a residual connection, and a multilayer perceptron (MLP) with two fully connected layers and GELU activation. Fig. 4 offers a visual representation of two successive Swin Transformer blocks applied in the context of the encoder. W-MSA limits attention computation to non-overlapping windows, which lowers the complexity from quadratic to linear regarding input dimensions. This approach improves computational efficiency and facilitates attention to local semantic structures. Conversely, the Shifted Window-based Multi-head Self-Attention (SW-MSA) increases the receptive field by incorporating a shift in the divided windows, enabling cross-window interactions. The alternation between W-MSA and SW-MSA in consecutive layers achieves a balance between local and global attention, leading to enhanced spatial awareness and semantic coherence.

Mathematically, one Swin Transformer block with input ${\textbf{z}}^{(\ell -1)}$ is specified by the following recursive operations:

1 $$\begin{aligned} \hat{{\textbf{z}}}^{(\ell )}= & \mathrm {W\text {-}MSA}\left( \textrm{LN}({\textbf{z}}^{(\ell -1)})\right) + {\textbf{z}}^{(\ell -1)} \end{aligned}$$

2 $$\begin{aligned} {\textbf{z}}^{(\ell )}= & \textrm{MLP}\left( \textrm{LN}(\hat{{\textbf{z}}}^{(\ell )})\right) + \hat{{\textbf{z}}}^{(\ell )} \end{aligned}$$

3 $$\begin{aligned} \hat{{\textbf{z}}}^{(\ell +1)}= & \mathrm {SW\text {-}MSA}\left( \textrm{LN}({\textbf{z}}^{(\ell )})\right) + {\textbf{z}}^{(\ell )} \end{aligned}$$

4 $$\begin{aligned} {\textbf{z}}^{(\ell +1)}= & \textrm{MLP}\left( \textrm{LN}(\hat{{\textbf{z}}}^{(\ell +1)})\right) + \hat{{\textbf{z}}}^{(\ell +1)} \end{aligned}$$

where ${\textbf{z}}^{(\ell )}$ is the output of the $\ell$-th MLP module and $\hat{{\textbf{z}}}^{(\ell )}$ is the intermediate result of the (S)W-MSA operation. The self-attention mechanism takes the standard scaled dot-product attention form^24,25^:

5 $$\begin{aligned} \textrm{Attention}({\textbf{Q}}, {\textbf{K}}, {\textbf{V}}) = \textrm{Softmax}\left( \frac{{\textbf{Q}} {\textbf{K}}^{\top }}{\sqrt{d}} + {\textbf{B}}\right) {\textbf{V}}, \end{aligned}$$

where ${\textbf{Q}}, {\textbf{K}}, {\textbf{V}} \in {\mathbb {R}}^{M^2 \times d}$ are the query, key, and value matrices, respectively. The feature size is denoted by *d*, $M^2$ denotes the number of patches in a window, and ${\textbf{B}}$ is the relative positional encoding bias matrix sampled from ${\mathbb {R}}^{(2M-1)\times (2M-1)}$.

### Feature enhancement unit

**Table 1:** Feature map dimensions and channel configurations at each Feature Enhancement Unit (FEU) stage.

FEU stage	Spatial resolution	CNN feature dims (h)	Transformer feature dims (g)	Output dims
FEU-1	112$\times$112	128	128	128
FEU-2	56$\times$56	256	256	256
FEU-3	28$\times$28	512	512	512
FEU-4	14$\times$14	768	768	768

**Fig. 5:**
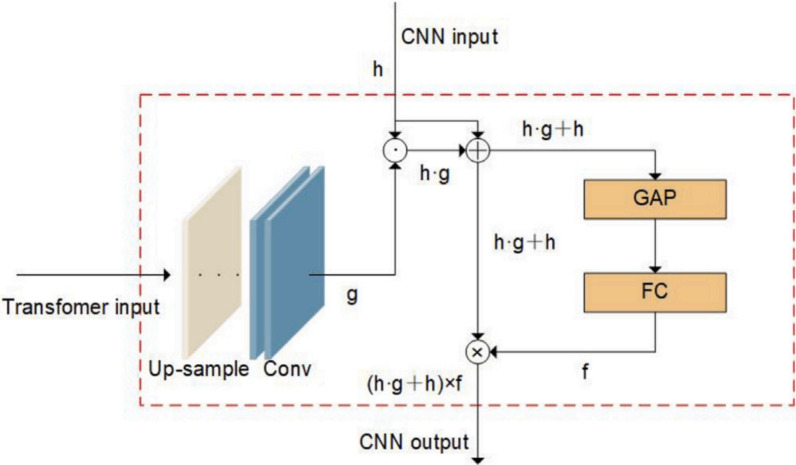
Structure of the Feature Enhancement Unit (FEU). The unit fuses upsampled transformer features with CNN features through multiplicative interaction, followed by global average pooling and channel-wise attention for refined enhancement.

The feature enhancement unit (FEU) serves as a bridge between the transformer pathway and the convolutional stream. Motivated by attention-based feature refinement mechanisms^39,40^, the FEU selectively transfers globally aggregated features from the transformer to the convolutional encoder. As depicted in Fig. 5, this unit takes intermediate feature maps from the transformer’s convolution module and from the CNN downsampling pathway. Let ${\textbf{g}}$ be the output of the transformer’s convolutional encoder upsampled with nearest-neighbor interpolation, and ${\textbf{h}}$ be the feature output of the downsampling module of the CNN. Element-wise multiplication is used to apply multiplicative fusion as ${\textbf{g}} \cdot {\textbf{h}} \cdot {\textbf{h}}$. This interaction results in the attention intermediate map. To further enrich these maps, a global average pooling layer along with a fully connected (FC) layer with sigmoid activation produces a vector ${\textbf{f}}$, which is broadcasted along channels. The FEU’s final output is computed by applying this learned weight vector to every channel:

6 $$\begin{aligned} Attention Output = ({\textbf{g}} \cdot {\textbf{h}} \cdot {\textbf{h}}) \times {\textbf{f}}, \end{aligned}$$

This output is then passed to the convolutional module in the CNN pathway. Through the use of four FEUs throughout the network, the architecture creates a consistent information flow between global semantic descriptors and localized features. This architecture enables strong attention-guided amplification at various stages and resolutions. As illustrated in Table 1, four FEUs are strategically placed at each encoder downsampling stage. At FEU-1, transformer features of spatial resolution 112$\times$112 with 128 channels are upsampled using nearest-neighbor interpolation to match the CNN feature maps of identical dimensions. Similarly, FEU-2, FEU-3, and FEU-4 operate at progressively reduced spatial resolutions of 56$\times$56, 28$\times$28, and 14$\times$14 with channel dimensions of 256, 512, and 768 respectively. At each FEU stage, the transformer output g is first upsampled to match the spatial dimensions of CNN feature map h. The multiplicative interaction (g$\cdot$h$\cdot$h) produces an attention intermediate map of identical dimensions to h, which is then refined through global average pooling and a fully connected layer to produce channel-wise weights f. The final FEU output, computed as (g$\cdot$h$\cdot$h)$\times$f, is passed to the subsequent CNN convolutional module, ensuring consistent feature dimensions throughout the encoding pathway

### Loss function

To train the segmentation model, a compound loss function of cross-entropy and Dice loss is used. The cross-entropy (CE) loss is a conventional metric that penalizes the difference between predicted probabilities and ground truth labels. It is formulated as:

7 $$\begin{aligned} {\mathscr {L}}_{\textrm{CE}} = -y\log (p) - (1 - y)\log (1 - p), \end{aligned}$$

where $y \in \{0,1\}$ is the ground truth label and $p \in [0,1]$ is the predicted probability for the foreground class. Because of the shape complexity and variability of anatomical structures in medical images, pixel-wise classification may not be adequate. The Dice loss is therefore introduced to account for class imbalance and boundary uncertainty. The Dice loss is given by:

8 $$\begin{aligned} {\mathscr {L}}_{\textrm{Dice}} = 1 - \frac{2 \sum _{j=1}^{N} p_j y_j}{\sum _{j=1}^{N} p_j^2 + \sum _{j=1}^{N} y_j^2}, \end{aligned}$$

where *N* is the total number of pixels, $p_j$ is the predicted probability, and $y_j$ is the binary ground truth at position *j*. The overall loss function optimized is a weighted sum of these two terms:

9 $$\begin{aligned} {\mathscr {L}}_{\textrm{Total}} = \alpha {\mathscr {L}}_{\textrm{CE}} + \beta {\mathscr {L}}_{\textrm{Dice}}, \end{aligned}$$

where the hyperparameters $\alpha$ and $\beta$ control the contribution of each loss term. In our implementation, we set these values to $\alpha = 0.5$ and $\beta = 1.0$, trading off classification performance with shape preservation.

## Experiments and results

This section provides an exhaustive account of the datasets, preprocessing methods, experimental settings, and performance metrics adopted to evaluate the proposed ResTransUNet model. We outline the training and test datasets, discuss image normalization and augmentation, and specify the evaluation metrics used for comparing model results. A set of ablation studies and comparison experiments with state-of-the-art methods are performed to measure the impact of each design element, followed by interpretability investigations and further validation on multi-organ segmentation tasks.

### Datasets

#### LiTS

The Liver Tumor Segmentation (LiTS) dataset^41^ was chosen as the main benchmark for training and testing of models. Published as part of the ISBI 2017 and MICCAI 2017 Liver Tumor Segmentation Challenge, the dataset consists of a training set of 131 CT volumes and a test set of 70 CT scans. The scans have between 42 and 1026 axial slices each, all resampled to an in-plane resolution of $512 \times 512$ pixels, with inter-slice distances varying from 0.45 to 6.0 mm. From the 131 available training volumes, we used 105 for training, 13 for validation, and 13 for testing using a fixed random split. The official LiTS test set of 70 volumes was not used as it requires online submission to the challenge server. All baseline comparisons were reproduced under the same data split and preprocessing protocol. This large variability in the number of slices and slice spacing makes this dataset well-suited for assessing segmentation robustness to varying imaging conditions.

#### 3D-IRCADb

The 3D Image Reconstruction for Algorithm Comparison Database (3D-IRCADb) is used for external validation. It offers contrast-enhanced CT scans of 20 patients, both males and females, with liver tumors in 75% of the patients. The dataset is further divided into two parts: 3D-IRCADb-01 contains 3D liver CT volumes of the abdomen, whereas 3D-IRCADb-02 consists of chest and abdominal scans of two anonymous patients. All scans have an axial resolution of $512 \times 512$ pixels. In spite of this, a few cases include low-contrast areas and overlapping structures, creating further segmentation challenges.

#### CHAOS

The Combined Healthy Abdominal Organ Segmentation (CHAOS) dataset^42^ contains CT and MRI scans. We limit the evaluation in this study to CT scans, which have annotations for the liver, kidneys, and spleen. The availability of multi-organ annotations allows us to test the generalization capability of the proposed model under real-world diagnosis scenarios.

#### SLIVER07

The Segmentation of the Liver Competition 2007 (SLIVER07) dataset^43^ is comprised of 30 annotated CT volumes, 20 of which are utilized for training and 10 for testing. The resolution of the slices is kept at $512 \times 512$ voxels, with the number of slices per volume varying from 64 to 502. The inter-slice distance ranges between 0.7 mm and 5.0 mm, and the intra-slice voxel spacing is in the range of 0.56 mm $\times$ 0.56 mm to 0.86 mm $\times$ 0.86 mm. This dataset provides accurate ground truth annotations from expert radiologists and serves as a solid benchmark for liver segmentation.

**Fig. 6:**
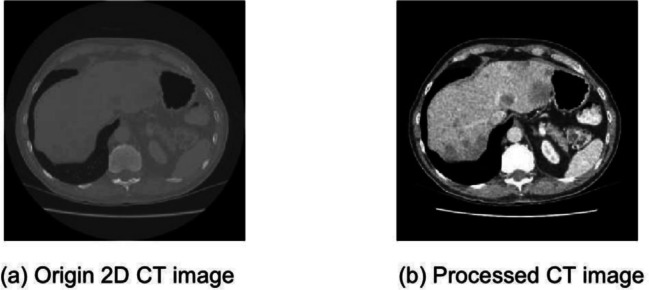
Comparison of original and preprocessed CT slices. The preprocessing pipeline enhances liver visibility by applying intensity windowing and normalization, improving edge clarity and regional contrast for segmentation.

### Augmentation and preprocessing

All CT volumes were first processed using a Hounsfield Unit (HU) window ranging from $-200$ to 200 to suppress non-relevant tissues and enhance liver boundaries. The HU-filtered voxel intensities were then linearly normalized to the range $[-1, 1]$ for consistency across patients. Figure 6 illustrates a visual comparison between the original and preprocessed slices, highlighting improved liver contrast and boundary sharpness. Then, the volumes were cropped and resized to $224 \times 224$ pixels to match the model input size and remove extraneous background. To increase the training distribution’s diversity and generalizability, we performed online augmentation methods such as horizontal and vertical flipping, random affine transforms, and rotations between $[-15^{\circ }, 15^{\circ }]$.

**Table 2:** System specifications used for model training and testing.

Component	Specification
Graphics Processing Unit (GPU)	NVIDIA RTX A6000
System Memory (RAM)	256 GB
Operating platform	Ubuntu 20.04
Storage capacity	4 TB
Development framework	PyTorch v1.11, Python v3.9

**Table 3:** Configuration of hyperparameters used in the training phase.

Parameter	Assigned value
Initial learning rate	0.0001
Mini-batch size	4
Number of Epochs	200 (with early stopping )
Optimization algorithm	Adam

### Experimental environment and parameters

All experiments were run on a system with an NVIDIA RTX A6000 GPU, 256 GB RAM, and Intel Xeon processors. Implementation was done in PyTorch. Table 2 provides the hardware and software specifications, and Table 3 provides the hyperparameter settings. The Adam optimizer was employed with an initial learning rate of $1 \times 10^{-4}$ and batch size of 4. The model was trained for 200 epochs with early stopping based on validation Dice score.

### Evaluation metrics

Three common metrics were used to assess segmentation quality: Dice Similarity Coefficient (DSC), Volume Overlap Error (VOE), and Relative Volume Difference (RVD). Let ${\mathscr {Y}}_{\text {gt}}$ be the ground truth mask and ${\mathscr {Y}}_{\text {pr}}$ be the predicted mask.

1. Dice Similarity Coefficient (DSC)
  10 $$\begin{aligned} \textrm{DSC}({\mathscr {Y}}_{\text {gt}}, {\mathscr {Y}}_{\text {pr}}) = \frac{2|{\mathscr {Y}}_{\text {gt}} \cap {\mathscr {Y}}_{\text {pr}}|}{|{\mathscr {Y}}_{\text {gt}}| + |{\mathscr {Y}}_{\text {pr}}|} \end{aligned}$$
  This measure calculates spatial overlap among high-confidence predicted and reference areas. high-confidence.
2. Volume Overlap Error (VOE)
  11 $$\begin{aligned} \textrm{VOE}({\mathscr {Y}}_{\text {gt}}, {\mathscr {Y}}_{\text {pr}}) = 1 - \frac{|{\mathscr {Y}}_{\text {gt}} \cap {\mathscr {Y}}_{\text {pr}}|}{|{\mathscr {Y}}_{\text {gt}} \cup {\mathscr {Y}}_{\text {pr}}|} \end{aligned}$$
  VOE calculates the ratio of disagreement in segmented volumes.
3. Relative Volume Difference (RVD)
  12 $$\begin{aligned} \textrm{RVD}({\mathscr {Y}}_{\text {gt}}, {\mathscr {Y}}_{\text {pr}}) = \frac{|{\mathscr {Y}}_{\text {pr}}| - |{\mathscr {Y}}_{\text {gt}}|}{|{\mathscr {Y}}_{\text {gt}}|} \end{aligned}$$
  RVD assesses volumetric discrepancy between ground truth and prediction.

**Table 4:** Component-wise ablation evaluation of ResTransUNet using the LiTS dataset. Each configuration tests the impact of selectively enabling architectural modules.

U-Net	Res-SE	Transformer	FEU	ASPP	DSC	VOE	RVD
$\checkmark$					0.9233 ± 0.081	0.1267 ± 0.116	$-0.0039$ ± 0.316
$\checkmark$	$\checkmark$				0.9292 ± 0.073	0.1154 ± 0.106	0.0413 ± 0.2415
$\checkmark$		$\checkmark$			0.9320 ± 0.071	0.1061 ± 0.105	0.0379 ± 0.3112
$\checkmark$	$\checkmark$	$\checkmark$		$\checkmark$	0.9301 ± 0.083	0.1079 ± 0.115	0.0324 ± 0.2222
$\checkmark$	$\checkmark$	$\checkmark$			0.9338 ± 0.113	0.1055 ± 0.132	0.0251 ± 0.0120
$\checkmark$	$\checkmark$	$\checkmark$	$\checkmark$		0.9472 ± 0.000	0.0981 ± 0.001	0.0164 ± 0.001
$\checkmark$	$\checkmark$	$\checkmark$	$\checkmark$	$\checkmark$	0.9535 ± 0.045	0.0804 ± 0.068	–0.0007 ± 0.095

### Ablation analysis

**Fig. 7:**
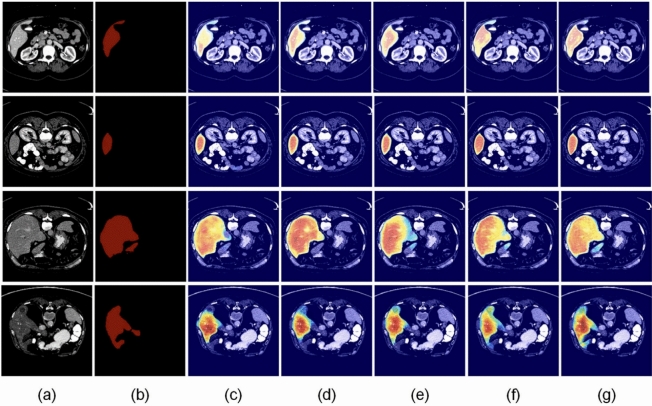
Grad-CAM-based visualization of model attention for different architectural configurations. The heatmaps highlight areas of high focus, with the full model displaying greater boundary attention and internal consistency.

An extensive ablation study was conducted to ascertain the contributions of major architectural elements, namely the Swin Transformer block and the Feature Enhancement Unit (FEU). The results, as evidenced in Table 4, demonstrate performance deterioration when either module is removed, thus validating their significant contribution towards improving segmentation accuracy. In addition, we utilized Grad-CAM to visualize the attention maps of various configurations, as indicated in Fig. 7. High activation regions are outlined in red. The visualizations validate that the complete model is successfully emphasizing target boundaries and organ interiors, while the baseline does not have such specificity.

**Fig. 8:**
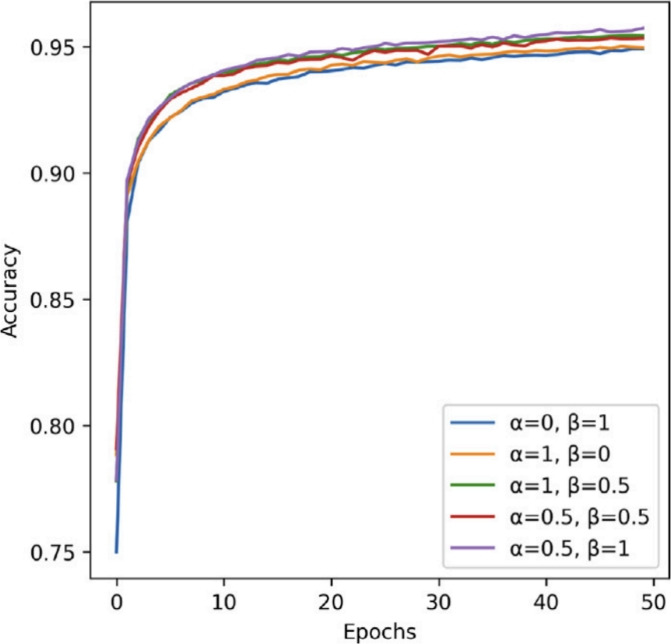
Effect of varying the weighting coefficients in the composite loss function on model accuracy. The graph illustrates segmentation performance across different combinations of $\alpha$ and $\beta$, revealing that balanced weighting leads to optimal Dice score.

**Fig. 9:**
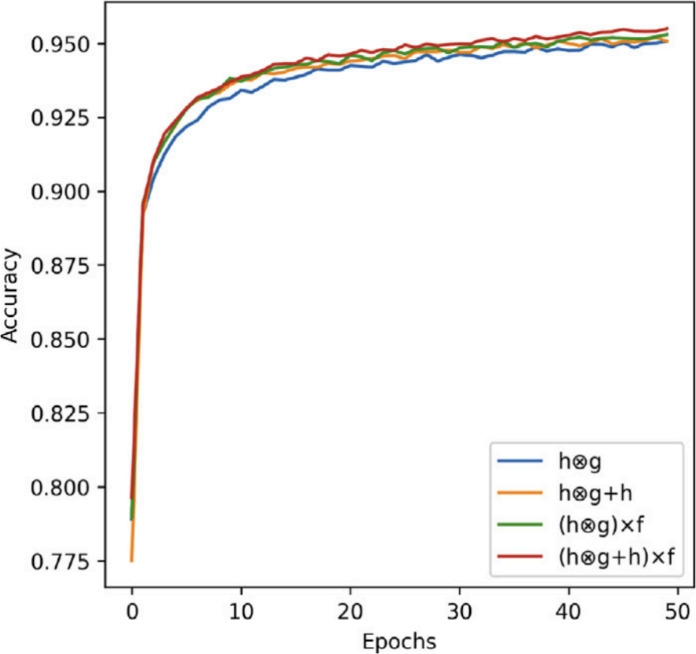
Comparative performance of various configurations of the Feature Enhancement Unit (FEU). The curves demonstrate how different structural modifications–such as removing residual connections or SE operations–affect segmentation accuracy and convergence.

To study the impact of changing the loss weights $\alpha$ and $\beta$ in Eq. (8), we have performed several experiments over combinations in the range (0, 1], and visualized the Dice scores in Fig. 8. The best performance was obtained with $\alpha = 0.5$ and $\beta = 1.0$, and these were then used in all the following experiments. Also, we conducted a structural ablation of the FEU as shown in Fig. 9. The comparison also involves versions where residual connections or SE blocks were skipped. Our suggested approach $({\textbf{g}} \cdot {\textbf{h}} \cdot {\textbf{h}}) \times {\textbf{f}}$ has the best accuracy and convergence rate, justifying the value of both multiplicative interaction and attentional fusion.

**Table 5:** Performance comparison of state-of-the-art segmentation models on the LiTS dataset. Results are reported as mean ± standard deviation.

Method	Dice (%)	VOE (%)	RVD (%)	Precision (%)	Recall (%)	*p*-value (Dice)
U-Net^7^	0.9233 ± 0.081	0.1267 ± 0.116	$-0.0039$ ± 0.316	0.9160 ± 0.094	0.9487 ± 0.087	$1.29 \times 10^{-13}$
U-Net++^10^	0.9347 ± 0.071	0.1061 ± 0.105	0.0215 ± 0.312	0.9391 ± 0.080	0.9496 ± 0.083	$3.18 \times 10^{-7}$
SAR-U-Net^30^	0.9389 ± 0.102	0.0976 ± 0.121	0.0112 ± 0.125	0.9451 ± 0.080	0.9513 ± 0.114	$2.54 \times 10^{-6}$
TransUNet^25^	0.9448 ± 0.060	0.0921 ± 0.087	0.0051 ± 0.134	0.9452 ± 0.067	0.9586 ± 0.069	$7.64 \times 10^{-3}$
SwinUNet^26^	0.9471 ± 0.059	0.0891 ± 0.085	0.0034 ± 0.142	0.9454 ± 0.062	0.9615 ± 0.072	$4.83 \times 10^{-3}$
GCHA-Net^17^	0.9464 ± 0.055	0.0918 ± 0.087	$-0.0153$ ± 0.120	0.9371 ± 0.078	0.9604 ± 0.054	$2.18 \times 10^{-3}$
RMAU-Net^18^	0.9521 ± 0.046	0.0819 ± 0.070	$-0.0040$ ± 0.089	0.9488 ± 0.062	0.9631 ± 0.046	$2.19 \times 10^{-2}$
Eres-UNet++^31^	0.9490 ± 0.054	0.0869 ± 0.082	$-0.0021$ ± 0.112	0.9463 ± 0.064	0.9627 ± 0.062	$1.39 \times 10^{-3}$
Proposed (Ours)	0.9535 ± 0.045	0.0804 ± 0.068	–0.0007 ± 0.095	0.9502 ± 0.053	0.9661 ± 0.056	–

**Table 6:** Comparative evaluation of segmentation models on the 3D-IRCADb dataset. Results are expressed as mean ± standard deviation.

Model	Dice (%)	VOE (%)	RVD (%)	Precision (%)	Recall (%)	*p*-value (Dice)
U-Net^7^	0.9278 ± 0.064	0.1206 ± 0.097	$-0.0144$ ± 0.117	0.9214 ± 0.087	0.9506 ± 0.063	$1.53 \times 10^{-16}$
U-Net++^10^	0.9253 ± 0.083	0.1119 ± 0.115	0.0509 ± 0.222	0.9437 ± 0.069	0.9299 ± 0.106	$2.33 \times 10^{-12}$
SAR-U-Net^30^	0.9318 ± 0.070	0.1077 ± 0.099	0.0077 ± 0.039	0.9508 ± 0.078	0.9270 ± 0.102	$4.53 \times 10^{-9}$
TransUNet^25^	0.9435 ± 0.067	0.0938 ± 0.079	0.0212 ± 0.116	0.9567 ± 0.065	0.9562 ± 0.058	$3.10 \times 10^{-7}$
SwinUNet^26^	0.9492 ± 0.055	0.0892 ± 0.077	0.0108 ± 0.096	0.9556 ± 0.074	0.9633 ± 0.055	$2.64 \times 10^{-4}$
GCHA-Net^17^	0.9586 ± 0.029	0.0626 ± 0.049	$-0.0090$ ± 0.066	0.9615 ± 0.047	0.9737 ± 0.033	$7.95 \times 10^{-3}$
RMAU-Net^18^	0.9622 ± 0.026	0.0602 ± 0.042	$-0.0057$ ± 0.063	0.9598 ± 0.047	0.9754 ± 0.036	$2.83 \times 10^{-3}$
Eres-UNet++^31^	0.9572 ± 0.037	0.0670 ± 0.072	0.0070 ± 0.077	0.9588 ± 0.055	0.9660 ± 0.037	$5.35 \times 10^{-3}$
Proposed (Ours)	0.9646 ± 0.023	0.0602 ± 0.040	–0.0034 ± 0.045	0.9614 ± 0.035	0.9767 ± 0.024	–

**Table 7:** Benchmark results of segmentation methods evaluated on the CHAOS dataset. Mean and standard deviation values are reported.

Model	Dice (%)	VOE (%)	RVD (%)	Precision (%)	Recall (%)	*p*-value (Dice)
U-Net^7^	0.9051 ± 0.066	0.1612 ± 0.098	0.0214 ± 0.143	0.9027 ± 0.047	0.9203 ± 0.097	$6.55 \times 10^{-16}$
U-Net++^10^	0.9181 ± 0.069	0.1510 ± 0.091	0.0213 ± 0.144	0.9160 ± 0.049	0.9182 ± 0.097	$3.11 \times 10^{-13}$
SAR-U-Net^30^	0.9400 ± 0.022	0.1216 ± 0.066	0.0077 ± 0.039	0.9439 ± 0.059	0.9511 ± 0.083	$9.48 \times 10^{-10}$
TransUNet^25^	0.9426 ± 0.035	0.1203 ± 0.064	0.0150 ± 0.035	0.9510 ± 0.033	0.9598 ± 0.042	$6.95 \times 10^{-7}$
SwinUNet^26^	0.9467 ± 0.021	0.1096 ± 0.043	0.0036 ± 0.083	0.9506 ± 0.047	0.9627 ± 0.051	$1.25 \times 10^{-4}$
GCHA-Net^17^	0.9268 ± 0.067	0.1313 ± 0.103	$-0.0150$ ± 0.305	0.9156 ± 0.122	0.9583 ± 0.077	$2.16 \times 10^{-3}$
RMAU-Net^18^	0.9521 ± 0.046	0.0923 ± 0.078	0.0040 ± 0.089	0.9488 ± 0.068	0.9631 ± 0.046	$1.32 \times 10^{-3}$
Eres-UNet++^31^	0.9513 ± 0.048	0.0803 ± 0.073	0.0103 ± 0.083	0.9522 ± 0.058	0.9591 ± 0.050	$1.81 \times 10^{-3}$
Proposed (Ours)	0.9549 ± 0.016	0.0788 ± 0.028	0.0026 ± 0.031	0.9538 ± 0.026	0.9643 ± 0.015	–

**Table 8:** Comparison of segmentation performance on the SLIVER07 dataset using various architectures.

Model	Dice (%)	VOE (%)	RVD (%)	Precision (%)	Recall (%)	*p*-value (Dice)
U-Net^7^	0.9056 ± 0.254	0.1585 ± 0.269	0.0273 ± 0.143	0.9204 ± 0.208	0.9270 ± 0.279	$1.55 \times 10^{-15}$
U-Net++^10^	0.9208 ± 0.229	0.1440 ± 0.320	0.0215 ± 0.144	0.9380 ± 0.256	0.9327 ± 0.312	$5.28 \times 10^{-12}$
SAR-U-Net^30^	0.9345 ± 0.090	0.1308 ± 0.233	0.0077 ± 0.039	0.9491 ± 0.199	0.9444 ± 0.232	$1.13 \times 10^{-7}$
TransUNet^25^	0.9393 ± 0.064	0.1202 ± 0.209	0.0150 ± 0.035	0.9450 ± 0.177	0.9443 ± 0.182	$4.11 \times 10^{-5}$
SwinUNet^26^	0.9461 ± 0.057	0.1123 ± 0.146	0.0036 ± 0.083	0.9476 ± 0.128	0.9610 ± 0.132	$1.73 \times 10^{-4}$
GCHA-Net^17^	0.9413 ± 0.070	0.1240 ± 0.140	$-0.0154$ ± 0.172	0.9432 ± 0.132	0.9581 ± 0.102	$4.83 \times 10^{-3}$
RMAU-Net^18^	0.9521 ± 0.046	0.0919 ± 0.076	0.0040 ± 0.089	0.9488 ± 0.062	0.9631 ± 0.046	$3.32 \times 10^{-3}$
Eres-UNet++^31^	0.9485 ± 0.137	0.1113 ± 0.160	0.0057 ± 0.142	0.9447 ± 0.096	0.9615 ± 0.056	$9.24 \times 10^{-3}$
Proposed (Ours)	0.9572 ± 0.180	0.0780 ± 0.091	0.0023 ± 0.031	0.9524 ± 0.154	0.9616 ± 0.191	–

### Comparison of models

The suggested approach was compared with eight state-of-the-art liver segmentation networks. The comparison results on the LiTS dataset, as presented in Table 5, show that ResTransUNet achieves the best DSC (0.9535), lowest VOE (0.0804), and least RVD ($-0.0007$), with precision (0.9614) and recall (0.9767). To evaluate cross-dataset generalization, we re-trained the model on LiTS and tested on 3D-IRCADb, CHAOS, and SLIVER07 datasets. The results, presented in Tables 6, 7, and 8, respectively, show systematic performance gains. The findings confirm the model’s robustness at segmenting liver anatomy across varying imaging conditions. All comparison results were run through a two-sided t-test to ensure statistical significance. All pairwise comparisons were evaluated using a two-sided paired t-test computed at the volume level, with each CT volume treated as an independent sample. Statistical tests were performed between the proposed method and each baseline using Dice scores obtained from the same test split (n=13 for LiTS). A p-value threshold of 0.05 was used to determine significance. The t-test yielded *p*-values less than 0.05, which ensured that our Dice score improvements did not arise from random chance.

### Model complexity and efficiency

**Fig. 10:**
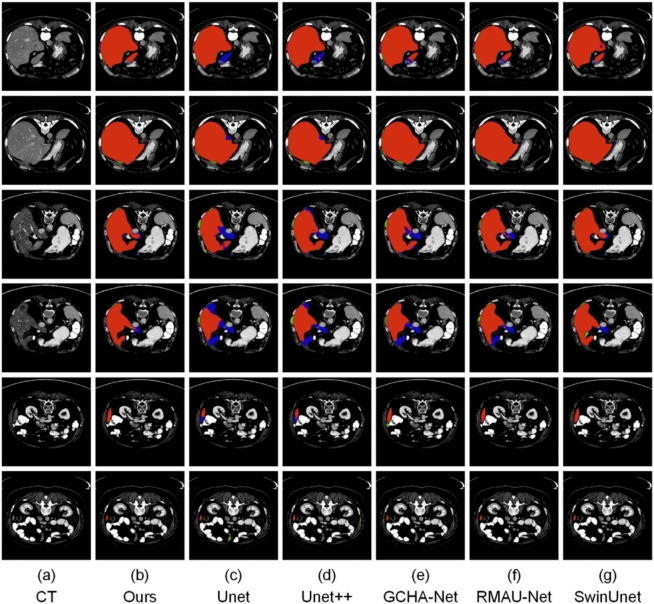
Visual comparison of segmentation results from various models on the LiTS dataset. Red regions denote correctly segmented liver areas (true positives), green areas represent over-segmented regions (false positives), and blue areas highlight missed regions (false negatives) in the predicted masks.

**Table 9:** Model efficiency summary, including parameter count, floating-point operations (FLOPs), and inference speed (FPS).

Architecture	Parameters (M)	FLOPs (G)	FPS
U-Net^7^	8.64	12.57	363
U-Net++^10^	36.63	106.11	56
TransUNet^25^	93.23	24.73	55
SwinUNet^26^	43.44	113.91	32
Proposed method	53.75	22.51	65

We also assessed the model’s efficiency by calculating the Frames Per Second (FPS), learnable parameters, and floating point operations (FLOPs). Table 9 indicates that although U-Net has the highest FPS, our model balances computational speed and segmentation quality. ResTransUNet has competitive FLOPs and parameters, coming in just behind U-Net and ahead of TransUNet and SwinUNet in overall efficiency. In order to check the generalizability of our model to more general clinical settings, we evaluated on the AbdomenCT-1k dataset^44^, shown in Table 10. The dataset contains segmentation tasks of organs including the liver, pancreas, and kidneys. The suggested model showed high segmentation accuracy for all types of organs. Especially, the model accurately dealt with challenging anatomical boundaries of the pancreas, which are conventionally hard to segment because of their size and shape variation.

**Fig. 11:**
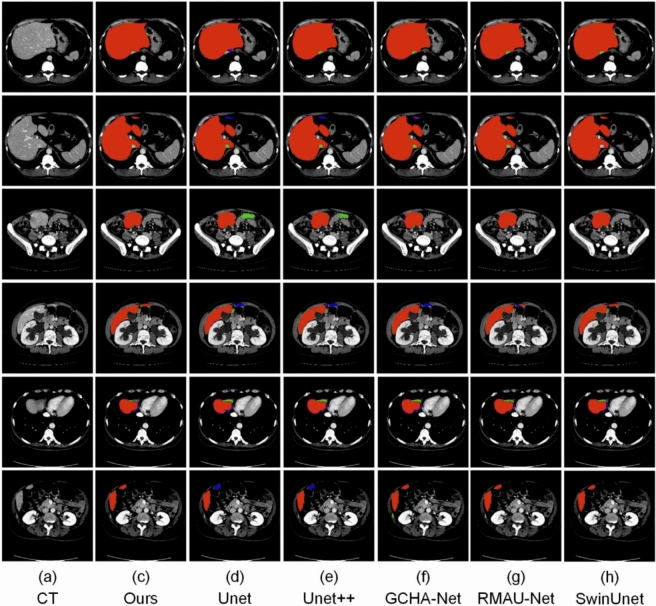
Visual comparison of segmentation results produced by different models on the 3D-IRCADb dataset. Red regions represent true positives, green areas indicate false positives (over-segmentation), and blue regions highlight false negatives (under-segmentation).

### Multi-organ segmentation generalizability

To further validate the generalizability of ResTransUNet beyond liver segmentation, we evaluated the model on the AbdomenCT-1K dataset across four organ categories: liver, kidney, spleen, and pancreas. As shown in Table 9, the proposed model achieves the highest Dice scores for liver (0.9551), spleen (0.9471), and pancreas (0.6589) compared to all baseline methods. The pancreas, which is particularly challenging due to its small size and high shape variability, showed a notable improvement over competing architectures, demonstrating the model’s capacity for fine-grained boundary delineation. These results confirm that the dual-encoder hybrid design of ResTransUNet generalizes effectively across diverse abdominal organ segmentation tasks.

### Visual segmentation results

**Fig. 12:**
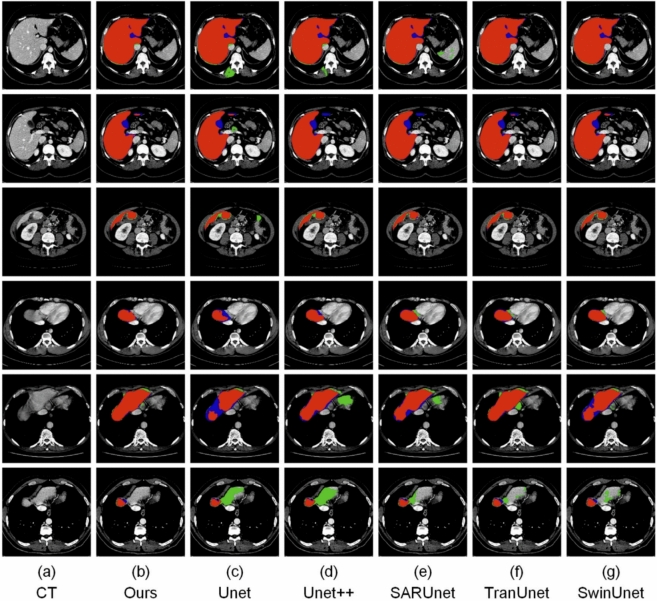
Visual comparison of segmentation results from various models on the CHAOS dataset. Red regions denote true positives, green indicates false positives (over-segmentation), and blue represents false negatives (under-segmentation).

**Fig. 13:**
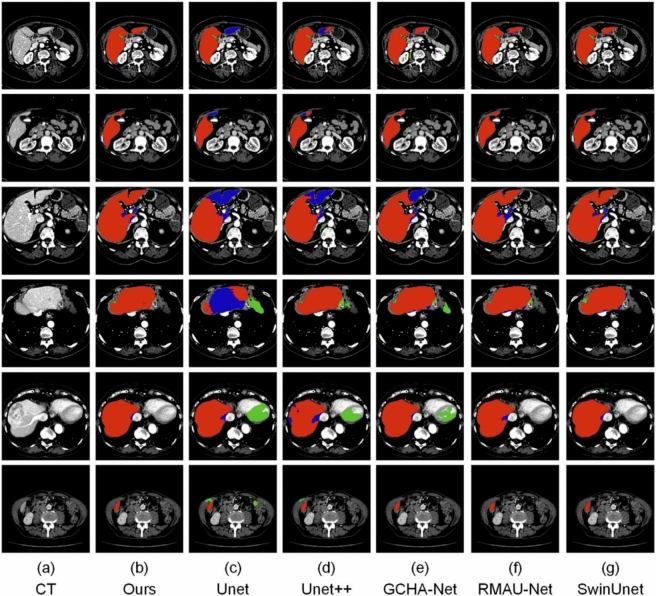
Visual comparison of segmentation results produced by different models on the SLIVER07 dataset. Red regions indicate true positives, green corresponds to false positives (over-segmentations), and blue denotes false negatives (under-segmentations).

To qualitatively assess segmentation performance, we show visual results in Figs. 10, 11, 12 and 13, comparing our approach with baseline architectures on four datasets. The visualizations illustrate better boundary adherence and fewer artifacts in our approach, especially for small and irregular liver areas. Although U-Net and U-Net++ sometimes under- or over-segment areas, our model ensures high fidelity in both common and difficult cases. However, edge segmentation in very low-contrast conditions is still an open challenge.

**Table 10:** Performance comparison of various segmentation networks on AbdomenCT-1k dataset across four organs. Metrics reported as mean ± standard deviation.

Method	Liver	Kidney	Spleen	Pancreas
U-Net^7^	0.9284 ± 0.127	0.6557 ± 0.453	0.8815 ± 0.198	0.6066 ± 0.378
U-Net++^10^	0.9373 ± 0.077	0.6722 ± 0.453	0.9011 ± 0.164	0.6163 ± 0.383
SAR-U-Net^30^	0.9467 ± 0.080	0.6782 ± 0.453	0.9093 ± 0.154	0.6357 ± 0.383
TransUNet^25^	0.9521 ± 0.078	0.6861 ± 0.450	0.9218 ± 0.214	0.6419 ± 0.364
SwinUNet^26^	0.9525 ± 0.071	**0.6927** ± **0.451**	0.9445 ± 0.204	0.6416 ± 0.376
GCHA-Net^17^	0.9545 ± 0.110	0.6857 ± 0.046	0.9449 ± 0.068	0.6480 ± 0.139
RMAU-Net^18^	0.9501 ± 0.081	0.6852 ± 0.047	0.9406 ± 0.070	0.6477 ± 0.121
Eres-UNet++^31^	0.9526 ± 0.078	0.6696 ± 0.450	0.9414 ± 0.127	0.6446 ± 0.307
Proposed Method	**0.9551 **± **0.078**	0.6887 ± 0.415	**0.9471** ± **0.186**	**0.6589 **± **0.385**

Bold value indicates statistically significant.

## Discussion

The ResTransUnet model demonstrates a remarkable breakthrough in the medical image segmentation paradigm through the development of a new architectural fusion between U-Net and transformer-based networks. The hybrid model is specifically designed to mitigate the inherent vulnerability of conventional U-Net architectures in modeling global contextual information, as well as pure transformer-based methods in preserving fine-grained spatial information owing to the lack of inductive biases. By the synergy of U-Net’s inductive bias in convolution and transformer pathway’s global attention, ResTransUnet possesses a good balance between local feature extraction and global semantic perception and generates highly precise segmentation output. The large-scale experimental study over popular benchmark datasets–namely, LiTS, 3D-IRCADb, CHAOS, and SLIVER07–secures the reality of ResTransUnet outperforming all the noticed metrics, namely, Dice Similarity Coefficient (DSC), Volumetric Overlap Error (VOE), and Relative Volume Difference (RVD). In particular, the model demonstrates great superiority in ensuring high precision and recall values even in challenging cases of complex anatomical variations or poor contrast boundaries. The incorporation of the transformer module, strategically positioned in the encoder series, dramatically improves the model’s capacity for capturing long-range dependencies and semantic context, enhancing U-Net’s capability for localization and producing more discriminative and rich feature maps.

Ablation study also attests to the usability of core architecture innovations, especially the incorporation of Swin Transformer block and Feature Enhancement Unit (FEU). The Swin Transformer block, with its hierarchical encoding and window-based multi-head self-attention, captures varied contextual clues and inter-region relations in the image, and FEU provides equivariant combination of convolutional and transformer features through adaptively amplifying task-specific spatial and channel-wise representations. Both modules allow ResTransUnet to surpass several state-of-the-art segmentation models by a significant margin, as certified by quantitative gains and visual comparisons. Apart from technical flawlessness, ResTransUnet is also bedeviled by enormous potential for clinical applications, specifically in liver segmentation procedures where speed and accuracy are paramount. The capacity to decrease the quantity of manual annotation and need for specialist intervention makes this model a viable option for optimizing diagnostic workflows, allowing early disease diagnosis, aiding in surgical planning in conditions such as hepatocellular carcinoma, and ultimately enhancing healthcare delivery and outcomes. However, despite how well the model generalizes across datasets and image conditions, scope for improvement cannot be ruled out. Despite the promising segmentation performance, several practical challenges must be addressed before clinical deployment. First, while ResTransUNet achieves 65 FPS on an NVIDIA RTX A6000 GPU, real-world hospital environments may rely on less powerful hardware, potentially limiting inference speed. Integration into existing PACS (Picture Archiving and Communication Systems) and radiology workflows would require standardized DICOM compatibility and minimal latency. Second, model robustness across different CT scanner manufacturers, acquisition protocols, and patient demographics needs further validation through prospective clinical trials. Finally, regulatory approval pathways (e.g., FDA clearance or CE marking) and clinician trust through model interpretability remain important considerations for practical adoptio. Future research can investigate hyperparameter optimization, alternative transformer structures, and advanced data augmentation techniques focused on improving robustness and transferability across domains. Further applications of ResTransUnet to other organ systems or multimodal imaging scenarios can further highlight its versatility and clinical utility. Together, the results establish ResTransUnet as an historic advance in medical image segmentation, bringing architectural elegance together with empirical effectiveness, laying a solid basis for future advances in computerized clinical imaging processing.

## Conclusion and future works

In this work, we presented a new deep learning architecture, ResTransUNet, specifically designed for automatic liver segmentation in CT images. The proposed architecture strategically integrates the capabilities of convolutional neural networks with the global modeling power of transformer-based pathways to overcome the inherent difficulties of medical image segmentation. At its foundation, ResTransUNet improves over the standard U-Net encoder by adding Squeeze-and-Excitation (SE) blocks within each convolutional unit, enabling the model to selectively focus on informative features and suppress irrelevant information. This attention-based refinement increases the model’s concentration on relevant anatomical boundaries and areas of interest. To additionally increase the receptive field and allow multi-scale feature extraction, Atrous Spatial Pyramid Pooling (ASPP) modules are used in transition and output layers, allowing the network to capture contextual information at different spatial resolutions. Additionally, residual connections are utilized to extend the network depth without compromising training stability, reducing the vanishing gradient threat and enhancing the flow of information throughout the model. One of the most significant contributions of our paper is the introduction of a transformer-based enhancement unit that complements each convolutional layer with global contextual representations. This module effectively consolidates local spatial features with long-range dependencies, thereby drastically increasing the representational capacity of the network and enhancing its segmentation accuracy in complicated medical imaging environments.

The efficacy and strength of ResTransUNet were verified on four benchmark datasets–LiTS2017, 3D-IRCADb, CHAOS, and SLIVER07–where the model consistently surpassed a number of state-of-the-art architectures on major performance indices, including Dice Similarity Coefficient (DSC), Volumetric Overlap Error (VOE), Relative Volume Difference (RVD), precision, and recall. Ablation experiments affirmed the vital roles played by each architectural innovation, particularly the Swin Transformer block and feature enhancement unit, in enhancing segmentation accuracy and generalizability. The model exhibited exceptional robustness in segmenting difficult areas like tiny lesions, vague boundaries, and low-contrast tissues, highlighting its readiness for real-world clinical use. Nevertheless, some drawbacks still remain–minor inconsistencies in segmentation were noticed around confusing boundary areas, sometimes leading to over- or under-segmentation. To overcome these difficulties, future efforts will involve the utilization of volumetric (z-axis) contextual information by expanding the framework into a full 3D architecture, injecting temporal coherence in sequential imaging, and investigating sophisticated data augmentation methods to enhance model robustness. Further optimization of hyperparameters and transformer settings may also provide performance enhancements and computational efficiency. ResTransUNet represents a solid advance in the domain of automated medical image segmentation, uniting local detail with global awareness to produce high-precision results across diverse anatomical and imaging conditions.

## Data Availability

The datasets used in this study are publicly accessible. The LiTS dataset is available at: https://competitions.codalab.org/competitions/17094. The 3D-IRCADb dataset can be accessed at: https://www.ircad.fr/research/data-sets/liver-segmentation-3d-ircadb-01/. The CHAOS dataset is available at: https://chaos.grand-challenge.org/. The SLIVER07 dataset can be accessed at: https://sliver07.grand-challenge.org/. The AbdomenCT-1K dataset is available at: https://github.com/JunMa11/AbdomenCT-1K. The code and scripts for this study are publicly available at: https://doi.org/10.5281/zenodo.19102606.
